# Characterising variability in youth mental health service populations: A detailed and scalable approach using digital technology

**DOI:** 10.1177/10398562231167681

**Published:** 2023-04-10

**Authors:** William Capon, Ian B Hickie, Sarah McKenna, Mathew Varidel, Matthew Richards, Haley M LaMonica, Daniel Rock, Elizabeth M Scott, Frank Iorfino

**Affiliations:** Brain and Mind Centre, 90098 The University of Sydney, Camperdown, NSW, Australia; WA Primary Health Alliance, Subiaco, WA, Australia; Brain and Mind Centre, 90098 The University of Sydney, Camperdown, NSW, Australia; Brain and Mind Centre, 4334 The University of Sydney, Camperdown, NSW, Australia

**Keywords:** youth mental health, digital technology, multidimensional assessment

## Abstract

**Objective:**

This study utilised digital technology to assess the clinical needs of young people presenting for care at *headspace* centres across Australia.

**Method:**

1490 young people (12–25 years) who presented to one of 11 *headspace* services from four geographical locations (urban New South Wales, urban South Australia, regional New South Wales, and regional Queensland) completed a digital multidimensional assessment at initial presentation. Characteristics were compared between services and geographical locations.

**Results:**

We identified major variation in the demographics, and the type and severity of needs across different services. Individuals from regional services were more likely to be younger, of Aboriginal and Torres Strait Islander origin, and present with psychotic-like symptoms and suicidality, while those in urban areas were more likely to have previously sought help and have problematic alcohol use. Further differences in age, distress, depressive symptoms, psychotic-like experiences, trauma, family history, alcohol use, education/employment engagement, and days out of role were identified between different urban sites.

**Conclusions:**

The variability between services provides insight into the heterogeneity of youth mental health populations which has implications for appropriate early intervention and prevention service provisions. We propose that integrating digital technologies has the potential to provide insights for smarter service planning and evaluation.

Youth mental health care is critical to prevent trajectories of lifelong morbidity and premature mortality.^1^ Consequently, governments interna-tionally have increased their investment in mental health services for young people. These services acknowledge the complexity, instability, and risks young people face as they transition to adulthood and navigate a period of major social, emotional, and biological change.

In Australia, *headspace* is a youth mental health initiative which has demonstrated the value of making access to services open and youth-friendly.^2–4^ These services improve engagement with evidence-based care for a heterogenous population of young people and is an international exemplar. Thus, it is important to understand the challenges that arise at a local level when these services are adopted in different communities. While the basic characteristics of this population are known,^2,5–7^ few studies have characterised the extent and variability of needs that arise by service and geographical area.

Digital technologies present the opportunity to assess clinical needs across multiple settings efficiently.^8^ Online assessments are standardised and scalable which facilitate the comprehensive assessment of needs appropriate to the specific risks and vulnerability in youth. This extends beyond measures of distress to functional impairment, comorbidity, and identifying at-risk mental states or behaviours for further assessment and intervention, based on a highly personalised and measurement-based youth mental health model.^9^

The purpose of this study is first to report on the variability in multidimensional needs of a cohort of *headspace* users in Australia between services and by geographical location. Second, we aim to demonstrate how digital technologies provide the opportunity to interrogate the needs of service populations at scale which can be used to inform service planning and provision.

## Methods

### Ethics

The Northern Sydney Local Health District Human Research Ethics Committees approved this study. All participants aged 14 and over gave online informed consent (via an opt out process).^10^ Parental consent was required for individuals aged 12–13 years.

### Participants

Participants were young people aged between 12 and 25 years who presented to *headspace* services^3^ between November 2018 and December 2021, and used the Innowell Platform.^10^

### The Innowell Platform

The Innowell Platform is a digital technology used clinically for the assessment, management, and monitoring of mental health and well-being.^11^ The web-based platform allows young people to complete a multidimensional clinical assessment at entry into care and over the course of care in a self-directed or clinically directed way. Results are displayed on a personalised dashboard to provide a better understanding of an individual’s needs, track progress, and get access to recommended self-directed or clinical care options. The tool is not intended as a crisis management tool, however, a standardised notification is used for those young people reporting suicidal thoughts and behaviours so that an immediate clinical response and protocol can be engaged by the service.

### Measures

The digital assessment includes mental health (psychological distress, depressed mood, anxiety, psychosis-like experiences, mania-like experiences, and post-traumatic stress), suicidal thoughts and/or behaviours, social and occupational functioning, sleep-wake cycle, social connectedness, alcohol use, tobacco use, self-harm, physical health, eating behaviours, and body image. Demographic information, and history of mental and physical health problems and treatment are also collected. Appendix 1 contains a detailed description of the assessment.

### Statistical analyses

All statistical analyses were conducted using R (version 4.2.1). We conducted three pairwise comparisons of clinical and demographic factors: (1) Urban South Australia (Urban SA) and Urban New South Wales (Urban NSW) (to compare within urban areas), (2) Regional Queensland (Regional QLD) and Regional New South Wales (Regional NSW) (to compare within regional areas), and (3) Urban and Regional sites (to compare between regions). Statistical methods were chosen for unequal groups.^9^ We used the Kruskal–Wallis test to compare continuous variables between each group, with post-hoc pairwise comparisons using Dunn’s test. For categorical variables, chi-squared tests were used. A Bonferroni correction was applied to control for the family wise error rate (*p* < .001). A heatmap was constructed for hierarchical clustering of variables and services (presented using a dendrogram). Binary variables were calculated as a proportion and continuous variables were scaled to a value between 0 and 1.

## Results

### Sample

A total of 1770 young people aged 12–25 years who attended *headspace* services consented for this study. Of the 1770 individuals, 1490 (84.2%) completed the initial questionnaire. The sample consisted of 277 from Urban South Australia, 948 from Urban NSW, 129 from Regional NSW, and 136 from Regional QLD (Table 1).

**Table 1.** Clinical and demographic characteristics of headspace users by geographical location

Characteristic	Number of individuals (*n* = 1490)				Comparison		
	Group						
	Urban SA (*n* = 277)	Urban NSW (*n* = 948)	Regional NSW (*n* = 129)	Regional QLD (*n* = 136)	Urban v regional	Urban SA v urban NSW	Regional QLD v regional NSW
Mean age (y), (SD)	19.1 (2.52)	20.2 (2.59)	19.0 (2.69)	17.6 (2.61)	***	***	***
Female at birth (%)	208 (75.1%)	671 (70.8%)	95 (73.6%)	91 (66.9%)			
English speaking (%)	267 (96.4%)	854 (90.1%)	127 (98.4%)	133 (97.8%)	***	**	
Indigenous status (%)	15 (5.4%)	28 (3.0%)	18 (14.0%)	25 (18.4%)	***		
Living alone (%)	9 (3.2%)	79 (8.3%)	4 (3.1%)	6 (4.4%)			
Single (%)	171 (61.7%)	602 (63.5%)	81 (62.8%)	99 (72.8%)			
*Social and occupational functioning*							
NEET (%) – missing 18	53 (19.1%)	70 (7.4%)	21 (16.3%)	21 (15.4%)	**	***	
Days out of role in last 30 days (SD) - missing 18	9.23 (8.61)	6.77 (7.49)	8.84 (8.73)	8.04 (8.39)		***	
WSAS total mean (SD) – missing 1	19.8 (8.38)	18.5 (8.20)	19.7 (7.37)	17.4 (8.71)		*	*
SSSS total mean (SD)	8.43 (2.90)	7.27 (2.82)	8.14 (3.01)	8.00 (2.94)	**		
Receiving government benefits (%)	86 (31.0%)	201 (21.2%)	45 (34.9%)	37 (27.2%)	*		
Independent support level (%)	85 (30.7%)	306 (32.3%)	40 (31.0%)	33 (24.3%)			
*Clinical presentation*							
Psychological distress (K-10 total mean)	34.4 (7.40)	31.5 (7.26)	33.3 (7.20)	32.6 (8.37)		***	
Depression (QIDS total mean) – missing 14	15.2 (4.80)	13.6 (4.82)	14.8 (4.87)	14.6 (4.77)	*	***	
Anxiety (OASIS total mean)	10.0 (4.29)	9.38 (4.07)	10.6 (4.28)	8.99 (4.07)		*	**
Psychosis (PQ16 total mean)	6.58 (3.93)	4.43 (3.55)	6.29 (4.19)	6.87 (4.05)	***	***	
Mania (ASRM total mean)	3.11 (2.92)	2.80 (2.86)	3.01 (2.76)	3.41 (3.16)	*		
Post-traumatic stress (PTSD5 total mean)	1.91 (2.05)	1.34 (1.82)	1.84 (2.05)	1.93 (2.03)	**	***	
Sleep-wake disturbances (%) – missing 7	166 (59.9%)	466 (49.2%)	69 (53.3%)	71 (52.2%)		**	
‘Probable’ eating disorder (%)	10 (3.6%)	40 (4.2%)	5 (3.9%)	6 (4.4%)			
*Personal history of mental illness (%)*							
Any family history – missing 16	222 (80.1%)	647 (68.2%)	93 (72.1%)	103 (75.7%)		***	
Sought prior treatment	193 (69.7%)	692 (73.0%)	84 (65.1%)	79 (58.1%)	***		
Experienced traumatic event	156 (56.3%)	421 (44.4%)	67 (51.9%)	76 (55.9%)		***	
Personal mental health problem – missing 1	209 (75.5%)	645 (68.0%)	94 (72.9%)	90 (66.2%)		*	
*Treatment utilisation (%)*							
Previous hospitalisation for mental health/behavioural problem – missing 1	29 (10.5%)	117 (12.3%)	19 (14.7%)	16 (11.8%)			
*Physical health comorbidities (%)*							
Any major physical illness	102 (36.8%)	296 (31.2%)	46 (35.7%)	38 (27.9%)			
*Alcohol and/or substance misuse (SD)*							
AUDIT-C total mean—missing 17	3.2 (2.6)	4.0 (2.6)	3.3 (2.8)	2.8 (3.0)	***	***
*Self-harm and suicidal thoughts and behaviours (SD)*							
SIDAS total mean	10.3 (11.3)	8.94 (11.5)	11.0 (11.8)	11.8 (11.9)	***	*	

*<.05, **<.01, ***<.001. Abbreviations: y, years; SD, standard deviation; NEET, not in education, employment, or training; WSAS, work and social adjustment scale; SSSS, Schuster’s social support scale; K-10, Kessler-10; QIDS, quick inventory of depressive symptomatology; OASIS, overall anxiety severity and impairment scale; PQ16, prodromal questionnaire; ASRM, Altman self-rating mania scale; PTSD, post-traumatic stress disorder; AUDIT-C, alcohol use disorders identification test.

### Cohort characteristics

Participants had a mean age of 19.6 years (median 20; SD = 2.72) and the majority (71.5%) were female at birth. Approximately 1 in 10 individuals were not involved in education, employment, or training (NEET; 11.2%), one-third were living independently (i.e. not financially supported by services, family and friends; 31.1%), and 5.6% identified as Aboriginal or Torres Strait Islanders. Most lived at home with family (81.4%) and were single (64.0%). Over two-thirds (68.0. %) were enrolled in education (e.g. TAFE, university and school). More than half of the cohort had previously sought help for mental illness (70.3%) and most had family history of mental illness (71.5%). Almost a third reported having an established physical illness (32.4%), and a quarter were receiving government benefits (24.8%).

The proportion of respondents reporting ‘moderate’ or higher psychological distress was 84.6% (Kessler-10 total ≥ 25; mean = 32.3 [SD = 7.47]), 75.7% for depression (Quick Inventory of Depressive Symptomatology total ≥ 11; mean = 14.1 [SD = 4.86]), and 87.9% for anxiety (Overall Anxiety Severity and Impairment Scale total ≥ 5; mean = 9.57 [SD = 4.15]).

### Comparing cohort characteristics between and within geographical locations

As shown in Table 1, compared to individuals from urban services, those at regional services were more likely to be younger (H = 81.66, *p* < .001), of Aboriginal or Torres Strait Islander origin (χ(1) = 62.46, *p* < .001), English speaking (χ(1) = 13.05, *p* < .001), have greater psychotic-like symptoms (H = 37.32, *p* < .001), and suicidality (H = 10.91, *p* < .001). Individuals at urban services were more likely to have sought previous help (χ(1) = 11.53, *p* < .001), and have more problems with alcohol use (H = 15.84, *p* < .001) compared to regional service youth.

Compared to Urban NSW, the Urban SA group had a greater proportion of those who were NEET (χ(1) = 31.80, *p* < .001), had higher psychological distress (z = 5.93, *p* < .001), depressive symptoms (z = 4.75, *p* < .001), psychotic-like experiences (z = 8.19, *p* < .001), post-traumatic stress (z = 4.32, *p* < .001), and were more likely to have experienced a traumatic event (χ(1) = 11.73, *p* < .001), have family history of mental illness (χ(1) = 14.14, *p* < .001), experience more days out of role in the last month (z = 4.35 *p* < .001), but were less likely to misuse alcohol (z = − 3.93, *p* < .001). Additionally, the regional NSW cohort was older than the regional QLD group (z = 3.89, *p* < .001).

## Discussion

This study compares the multidimensional needs of *headspace* users between different services and geographical regions. The findings reveal meaningful patterns of variability between services and provide insight into the heterogeneity of youth mental health populations. We propose that the smarter use of digital technologies within service infrastructures can facilitate real-time insights into the varying needs of specific populations.

Overall, the *headspace* cohort in this study had very high distress, moderate depression, and moderate anxiety. These levels of distress are slightly higher yet comparable with a recent *headspace* evaluation report, which is problematic given they found that most young people with higher levels of distress do not significantly improve according to the clinically significant change index for social and occupational outcomes.^4^ For example, improvements in social and occupational functioning were lowest for those with high distress.^4^ While high distress at entry may be associated with reductions in distress and symptoms over the course of care, these improvements do not necessarily translate to improvements in functional outcomes. This is consistent with previous work in a different headspace cohort illustrating similar rates of low improvement for those with more substantial needs.^12^ Furthermore, most young people in this sample had a familial history of mental illness (71.5%), and 11.2% were NEET, which are both independently associated with poor outcomes.^13,14^

**Figure 1.**
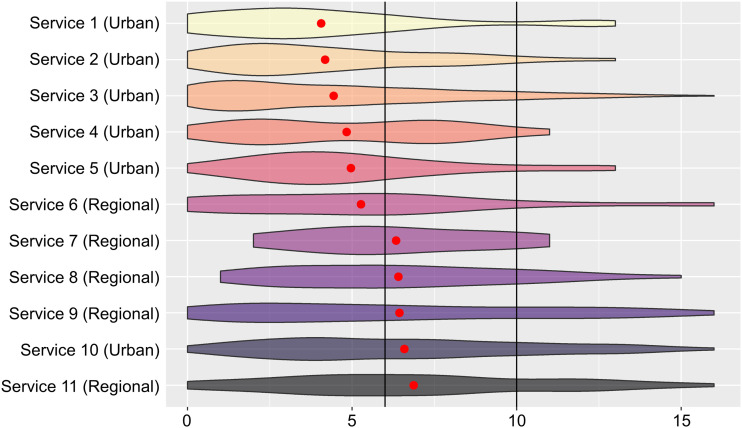
The distribution of individual scores for psychosis-like experiences measured by the prodromal questionnaire (PQ16) for 11 service locations. Vertical black lines indicate different thresholds for further assessment of a psychotic syndrome (6 indicates possible syndrome and 10 indicates a probable syndrome). Red dot represents mean scores for each service.

More specific analyses of needs across services have shown how patterns of risk and vulnerability emerge across different settings. Individuals attending regional services were more likely to be younger, of Aboriginal or Torres Strait Islander heritage, and have higher rates of suicidality and psychotic-like experiences (Figure 1). Aboriginal or Torres Strait Islander people are more likely to discontinue treatment,^6^ making them a priority group.^4^ Psychotic-like experiences are associated with a higher risk of illness progression^13^ and indicative of the need for further assessments and intervention from specialists which may not be available in regional and remote areas of Australia. The ostensibly more complex group of individuals attending regional *headspace* services was also younger than urban areas. Urban services may have the resources to direct similarly complex cases to specialised services, without the same access in regional areas. This lack of access to specialised care pathways, together with other psycho-socio-cultural factors that increase the risk of more severe illness, could partially explain the increased rates observed in the regional services relative to the urban services. Longer wait times and lower engagement in regional areas highlights the need for innovative approaches to facilitate engagement for these risk groups,^4^ including leveraging digital technologies to connect them to specialised support immediately.^15^

**Figure 2.**
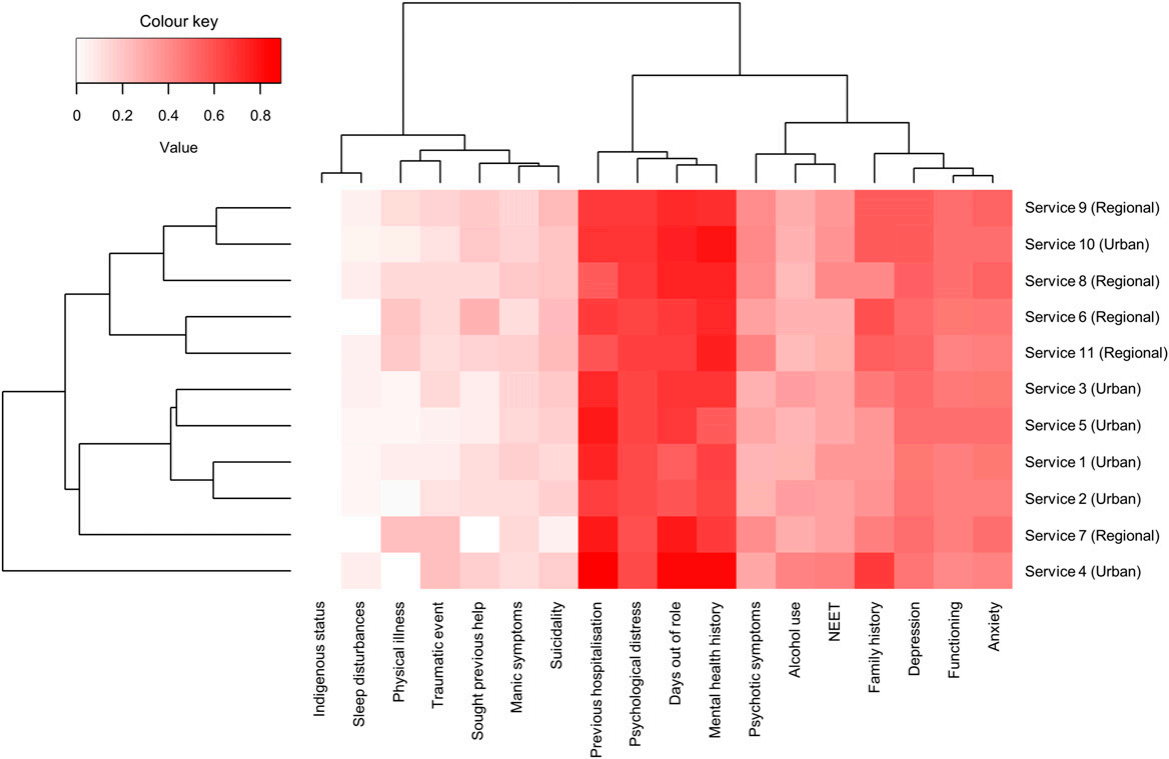
A heatmap of multiple clinical and demographic characteristics. Dendrogram indicates hierarchical clustering of variables and services. For binary variables, values are presented as a proportion from 0 to 1. For continuous variables, a relative scale score was generated by calculating the mean value of each group and converting it to a number between 0 and 1 using the following formula $r=\frac{\bar{x}-m}{M}$ where $\bar{x}$ is the mean, m is the minimum possible score, and M is the maximum possible score. Abbreviations: NEET = not in education, employment.

Compared with Urban NSW services, the Urban SA cohort were younger with less alcohol misuse but higher rates of distress, psychotic-like experiences, depression, days out of role, NEET, trauma, and family history. This distinction reiterates urbanicity does not completely explain the variation we observe and that there are many other clinical or psycho-socio-cultural factors (e.g. indigenous and migrant populations, socioeconomic status) at a specific local level that may be driving these differences. Figure 2 showcases service-to-service variability in factors that are crucial for predicting short- and long-term outcomes, such as functional impairment and at-risk mental states.^13^ This highlights the importance of monitoring unique population needs and developing local capacity to provide appropriate care.

The standardisation of a digital assessment platform which comprehensively interrogates key clinical characteristics according to a highly personalised and measurement-based care model of youth allows for an immediate understanding of need.^9^ This information can be used at a local level to inform decisions about service previsions as required to meet the needs of specific communities. While many health services currently collect data about their service population, we would argue that the broader use of a complete information and analytics feedback system for service planning is not common in mental health care. Such real-time data collection and monitoring can also be linked with other decision-support tools to forecast potential future needs based on current trends.^8^ The access to multidimensional data for Primary Health Networks has the potential to guide specialised resource allocation and the facilitation of care coordination.^16^*headspace* has demonstrated the capacity to absorb variation in needs which is both a benefit and vulnerability of this model. Yet, with smarter tools, funders of other locality-based services can reset their own service parameters knowing the role *headspace* takes within the context of a local healthcare eco-system and the needs of its population.^8,17^

The first limitation of this study is that this was not a systematic evaluation of the *headspace* population so sample sizes for each group were unequal and small for some services. Though this may limit the generalisability of these findings, this is the first study to examine the detailed variability across services which provides further information about specific clinical needs not available in larger evaluations. This work provides support for larger investigations using these methods. Second, it is unclear whether data were missing at random or if personal circumstances affected engagement. Those with incomplete secondary education, in the lowest income quintile, living in single person household, living with a disability, or are unemployed/not in the labour force are less likely to have access to digital technologies which could affect their capacity to engage. This emphasises the need for health policy and governments to ensure that communities have equitable access to this critical infrastructure. Third, we only investigated the initial presentation of individuals across services, and thus cannot infer about changing needs throughout care which should be a focus of future longitudinal studies.

## Conclusion

These findings quantify the variability in clinical needs for *headspace* clients and provide insight into how the broad availability of services influence local presentation patterns. We propose that the use and integration of digital technologies into youth mental health systems internationally is needed to gain major clinical insights about service populations, crucial for service planning and evaluation.

## Supplemental Material

Supplemental Material - Characterising variability in youth mental health service populations: A detailed and scalable approach using digital technology Click here for additional data file. Supplemental Material for Characterising variability in youth mental health service populations: A detailed and scalable approach using digital technology by William Capon, Ian B Hickie, Sarah McKenna, Mathew Varidel, Matthew Richards, Haley M LaMonica, Daniel Rock, Elizabeth M Scott and Frank Iorfino in Australasian Psychiatry

Supplemental Material - Characterising variability in youth mental health service populations: A detailed and scalable approach using digital technology Click here for additional data file. Supplemental Material for Characterising variability in youth mental health service populations: A detailed and scalable approach using digital technology by William Capon, Ian B Hickie, Sarah McKenna, Mathew Varidel, Matthew Richards, Haley M LaMonica, Daniel Rock, Elizabeth M Scott and Frank Iorfino in Australasian Psychiatry
