# Supplementary material for: Characterising variability in youth mental health service populations: A detailed and scalable approach using digital technology
Source: Australas Psychiatry. 2023 Apr 10;31(3):295–301. doi: 10.1177/10398562231167681 (PMC10293478; doi:10.1177/10398562231167681)
Supplement: Supplemental Material - Characterising variability in youth mental health service populations: A detailed and scalable approach using digital technology [file sj-pdf-1-apy-10.1177_10398562231167681.pdf]

Appendix 1 – Description of questionnaires in the Innowell Platform.

Distress:

K-10 – The Kessler-10 (K-10) measures psychological distress over the past 4 weeks [1]. It is a well-validated measure widely used in adult and adolescent populations in both clinical and community settings. The scale consists of 10 items with five multiple choice options ranging from "none of the time" (1) to "all of the time" (5). Total scores range from 10-50. A total score of under 20 indicates 'likely no distress', 20-24 indicates 'likely mild mental disorder', 25-29 indicates 'likely moderate mental disorder', over 30 indicates 'likely severe mental disorder' [2]. The K-10 has moderate reliability (kappa scores 0.42-0.74).

Suicidality:

SIDAS – The Suicide Ideation Attributes Scale (SIDAS) is a five-item scale assessing suicidal ideation over the past month. The scale assesses frequency, controllability, closeness to attempt, distress and interference with daily activities on a 10-point Likert scale (ranging from 0 ("never") to 10 ("always")). A total score of zero corresponds to "no current ideation", a score of one to 20 corresponds to "low current suicidal ideation", and a score of 21 to 50 corresponds to "high current suicidal ideation". The scale has strong internal reliability (Cronbach $\alpha = 0.91$).[3]

C-SSRS – The Columbia-Suicide Severity Rating Scale (C-SSRS) aims to measure suicidal thoughts and behaviours in the past month and entire lifetime. The questionnaire assesses four constructs: severity of ideation, intensity of ideation, suicidal behaviours, and lethality.[4]

Psychosis-like experiences:

PQ-16 – The Prodromal Questionnaire (PQ-16) is a self-report measure used to screen for individuals at risk of psychosis and select individuals for interview of psychosis risk [5]. It was adapted from the 92-item Prodromal Questionnaire, including nine items from the perceptual abnormalities subscale, five items from the unusual thought content/delusional ideas subscale, and two items from the negative symptoms subscale [6]. Questions require "True" or "False" answers to statements describing feelings, experiences, or symptoms of psychosis. In the event of a "True" response, the participant is required to then interpret the perceived distress of that on a Likert scale (0-3, with 3 being severe distress). A cut-off score of 6 or more on the symptom-scale has been shown to detect at-risk mental states with 87% specificity and sensitivity.[5]

Mania-like experiences

ASRM - The Altman Self-Rating Mania Scale (ASRM) is a 5-item scale used to assess the presence and/or severity of manic-like symptoms during the past 7 days. Each item is scored on a 5-point scale (0-4), with total scores ranging from 0-20 and higher scores indicating greater severity. A score greater than 5 indicates a high likelihood of a manic or hypomanic condition, as per 86% sensitivity and 87% sensitivity.[7]

Functioning

Youth not in education or employment (NEET) - Engagement with either employment, education, or training was based on questions from the Organisation of Economic Co-operation and Development (OECD), and Census of Population and Housing, Australian Bureau of Statistics (ABS) [8]. These four multiple choice questions were: 1) Are you currently engaged in education or study (e.g. school, TAFE or university) on a regular basis?; 2) Are you currently engaged in paid employment or work on a regular basis?; 3) Are you currently engaged in voluntary work through an organisation or group on a regular basis?; and 4) Are you currently providing unpaid care, help or assistance to family members or others because of a disability, a long term illness or problems related to old age on a regular basis? Individuals not involved in employment, education, or training were classified as NEET.

WSAS - The Work and Social Adjustment Scale (WSAS) is a brief and reliable measure of work and social adjustment. The questions aim to assess whether an individual is currently impaired or unable to perform day-to-day tasks, due to their mental health. The scale consists of five items that require the individual to rate between 0-8 ("not at all" to "very severely"), based on their agreement with the statement. A maximum total score is achieved by summing all 5 items. A total score greater than 20 suggests moderately severe psychopathology, 10-20 suggests significant functional impairment with less severe symptomatology, and scores under 10 are associated with subclinical populations. The scale has a test-retest correlation of 0.73 and has correlations of 0.76 for severity of depression and 0.61 for obsessive-compulsive disorder symptoms.[9]

Alcohol, Tobacco, Cannabis

ASSIST - The Alcohol, Smoking and Substance Involvement Screening Test (ASSIST) is an 8-item questionnaire that aims to detect substance use-related problems. It screens for use of tobacco, alcohol, cannabis, cocaine, amphetamine-type stimulants, sedatives and sleeping pills, hallucinogens, inhalants, opioids, and other drugs. Besides the first question which concerns life-time experiences, each question requires the individual to respond to questions that concern the prior 3-months on either 5- or 3- point Likert scales.[10]

AUDIT-C - The Alcohol Use Disorders Identification Test (AUDIT-C) is a brief measure consisting of three questions related to the frequency of general and binge alcohol consumption over the past year [11]. Each question has 5 options that increase in frequency or quantity of consumption (scores range from 0-4) [12]. A total score of 0-3 indicates low-risk drinking, 4-5 moderate risk, and scores greater than five indicates high risk drinking, however, this may not apply if total points come from q1 (i.e., when q2 and q3 =0).

Social connection

SSSS - The Schuster's Social Support Scale (SSSS) is a 6-item questionnaire that aims to assess the frequency of both supportive and negative interactions with family and friends [13]. The first five questions are scored on a 4-point Likert scale (0-3; "never" to "often") with alpha reliability ranging from 0.56-0.75, and the sixth question requires a Yes/No response.[13]

Depression

QIDS-SR - The Quick Inventory of Depressive Symptomatology – Self-report (QIDS-SR) consists of 16-items that aim to assess nine domains of depression during the preceding seven days [14]. These domains are related to the DSM-IV diagnosis of a major depressive disorder, including: sleep, mood, appetite/weight, concentration/decision making, self-view, suicidal ideation, general interest, energy level, and agitation. Each item is scored on a scale between 0-3 points and scoring instructions determine the total score (which ranges from 0-27).[15] Scores greater than 21 indicate very severe depression, 16-20 indicate severe depression, 11-15 indicate moderate depression, 6-10 indicate mild depression, and scores of 5 or lower indicate no depression.

Anxiety

OASIS - The Overall Anxiety Severity and Impairment Scale aims to assess the severity and impairment of anxiety-related symptoms over the past seven days [16]. The scale consists of five multiple choice questions with five options that are scored from 0-4, with higher scores indicating greater severity and/or impairment. A cut-off score of 8 has high validity (87%) for identifying anxiety disorders [17].

Physical health

Height, weight, and waist circumference

Body mass index (BMI) is calculated by Weight/(Height$^{\wedge 2}$) to estimate total body fat in proportion to total body weight. It is used to estimate risk of cardiovascular, metabolic, and other diseases. Waist circumference, however, is a more accurate estimate of visceral fat and more predictive of cardiovascular diseases. For women, a waist circumference of 80-87cm is considered increased risk, and 88+cm is greatly increased. For men, a waist circumference of 94-101cm is considered increased risk, and 102+cm is considered greatly increased risk.[18]

IPAQ - The International Physical Activity Questionnaire (IPAQ) aims to determine the average physical activity of an individual and can be scored on a continuous and/or categorial scale (high, moderate, or low) [19]. First, all activity is calculated in minutes. Second, minutes should be converted to metabolic equivalent of task (MET) minutes (multiply the minutes by the relevant scalar; walking =3.3, moderate activity=4, vigorous activity =8). Third, multiply MET minutes by number of days the activity was performed. For categorical scoring, high = over 3000 MET minutes a week OR over 1500 MET minutes per week with 3 or more days of vigorous exercise; moderate = at least 3 days of vigorous activity or walking of 30 or more mins per day, OR 5+ days of moderate intensity activity and/or walking (minimum 30 mins per day), OR 5+ days of walking, moderate intensity, or vigorous activity equating to 600+ MET minutes; and low = not meeting high or moderate.

Sleep-wake cycle

PSQI - The Pittsburgh Sleep Quality Index (PSQI) is a questionnaire that aims to assess sleep quality and disturbances over the past month [20]. It consists of nineteen-items that assess seven domains: sleep quality, sleep latency, sleep duration, habitual sleep efficiency, sleep disturbances, use of sleeping medication, and daytime dysfunction. Each of the seven component scores are summed together to form a global score [20].

MCTQ – The Munich Chronotype Questionnaire (MCTQ) is a self-report scale that assesses bed- and rise-times, and self-assessment of individual chronotype [21]. The chronotype options range from extremely early to extremely late and is determined by using the midpoint between onset and offset of sleep.

Post-traumatic stress

PC-PTSD-5 - The Primary Care PTSD Screen (PC-PTSD) is a five-item questionnaire that aims to assess PTSD symptoms which reflect the DSM-V diagnostic criteria [22]. Each item is scored as either Yes or No (1 or 0), and a maximum total score is 5. A cut-off of 3 is optimally sensitive (reduces false negatives; κ[1]) = 0.93, standard error = 0.041), yet a cut-off of 4 is optimally efficient (good balance between false positive and negatives; κ[0.5] = 0.63, standard error = 0.052) [22].

Eating behaviours and body image

EDE – The Eating Disorder Examination Questionnaire (EDE-Q) is based on the eating disorder examination interview and aims to assess eating behaviours and body image disturbance over the past four weeks [23]. The questionnaire uses a combination of Likert scales and Yes/No options.

REFERENCES

1. Kessler RC, Andrews G, Colpe LJ, Hiripi E, Mroczek DK, Normand SL, et al. Short screening scales to monitor population prevalences and trends in non-specific psychological distress. Psychol Med. 2002;32(6):959-76.

2. Andrews G, Slade T. Interpreting scores on the Kessler Psychological Distress Scale (K10). Aust N Z J Public Health. 2001;25(6):494-7.

3. van Spijker BA, Batterham PJ, Calear AL, Farrer L, Christensen H, Reynolds J, et al. The suicidal ideation attributes scale (SIDAS): Community-based validation study of a new scale for the measurement of suicidal ideation. Suicide Life Threat Behav. 2014;44(4):408-19.

4. Posner K, Brown GK, Stanley B, Brent DA, Yershova KV, Oquendo MA, et al. The Columbia-Suicide Severity Rating Scale: initial validity and internal consistency findings from three multisite studies with adolescents and adults. Am J Psychiatry. 2011;168(12):1266-77.

5. Ising HK, Veling W, Loewy RL, Rietveld MW, Rietdijk J, Dragt S, et al. The validity of the 16-item version of the Prodromal Questionnaire (PQ-16) to screen for ultra high risk of developing psychosis in the general help-seeking population. Schizophr Bull. 2012;38(6):1288-96.

6. Howie C, Hanna D, Shannon C, Davidson G, Mulholland C. The Structure of the Prodromal Questionnaire-16 (PQ-16): Exploratory and confirmatory factor analyses in a general non-help-seeking population sample. Early Interv Psychiatry. 2021.

7. Altman EG, Hedeker D, Peterson JL, Davis JM. The Altman Self-Rating Mania Scale. Biol Psychiatry. 1997;42(10):948-55.

8. Youth not in employment, education or training (NEET) [Internet]. 2017. Available from: https://www.oecd-ilibrary.org/content/data/72d1033a-en.

9. Mundt JC, Marks IM, Shear MK, Greist JH. The Work and Social Adjustment Scale: a simple measure of impairment in functioning. Br J Psychiatry. 2002;180:461-4.

10. Humeniuk R, Ali R, Babor TF, Farrell M, Formigoni ML, Jittiwutikarn J, et al. Validation of the Alcohol, Smoking And Substance Involvement Screening Test (ASSIST). Addiction. 2008;103(6):1039-47.

11. World Health Organisation. AUDIT: The Alcohol Use Disorders Identification Test: Guidelines for Use in Primary Care. 2 ed. Geneva2001.

12. Bush K, Kivlahan DR, McDonell MB, Fihn SD, Bradley KA. The AUDIT alcohol consumption questions (AUDIT-C): an effective brief screening test for problem drinking. Ambulatory Care Quality Improvement Project (ACQUIP). Alcohol Use Disorders Identification Test. Arch Intern Med. 1998;158(16):1789-95.

13. Schuster TL, Kessler RC, Aseltine RH, Jr. Supportive interactions, negative interactions, and depressed mood. Am J Community Psychol. 1990;18(3):423-38.

14. Rush AJ, Trivedi MH, Ibrahim HM, Carmody TJ, Arnow B, Klein DN, et al. The 16-Item Quick Inventory of Depressive Symptomatology (QIDS), clinician rating (QIDS-C), and self-report (QIDS-SR): a psychometric evaluation in patients with chronic major depression. Biol Psychiatry. 2003;54(5):573-83.

15. Brown ES, Murray M, Carmody TJ, Kennard BD, Hughes CW, Khan DA, et al. The Quick Inventory of Depressive Symptomatology-Self-report: a psychometric evaluation in patients with asthma and major depressive disorder. Ann Allergy Asthma Immunol. 2008;100(5):433-8.

16. Norman SB, Cissell SH, Means-Christensen AJ, Stein MB. Development and validation of an Overall Anxiety Severity And Impairment Scale (OASIS). Depress Anxiety. 2006;23(4):245-9.

17. Campbell-Sills L, Norman SB, Craske MG, Sullivan G, Lang AJ, Chavira DA, et al. Validation of a brief measure of anxiety-related severity and impairment: the Overall Anxiety Severity and Impairment Scale (OASIS). J Affect Disord. 2009;112(1-3):92-101.

18. Health Direct. Body mass index (BMI) and waist circumference 2020 [

19. Craig CL, Marshall AL, Sjostrom M, Bauman AE, Booth ML, Ainsworth BE, et al. International physical activity questionnaire: 12-country reliability and validity. Med Sci Sports Exerc. 2003;35(8):1381-95.

20. Buysse DJ, Reynolds CF, 3rd, Monk TH, Berman SR, Kupfer DJ. The Pittsburgh Sleep Quality Index: a new instrument for psychiatric practice and research. Psychiatry Res. 1989;28(2):193-213.

21. Roenneberg T, Wirz-Justice A, Merrow M. Life between clocks: daily temporal patterns of human chronotypes. J Biol Rhythms. 2003;18(1):80-90.

22. Prins A, Bovin MJ, Smolenski DJ, Marx BP, Kimerling R, Jenkins-Guarnieri MA, et al. The Primary Care PTSD Screen for DSM-5 (PC-PTSD-5): Development and Evaluation Within a Veteran Primary Care Sample. J Gen Intern Med. 2016;31(10):1206-11.

23. Hay PJ, Mond J, Buttner P, Darby A. Eating disorder behaviors are increasing: findings from two sequential community surveys in South Australia. PLoS One. 2008;3(2):e1541.
